# The Functional Significance of Posttranslational Modifications on Polo-Like Kinase 1 Revealed by Chemical Genetic Complementation

**DOI:** 10.1371/journal.pone.0150225

**Published:** 2016-02-26

**Authors:** Amber L. Lasek, Brittany M. McPherson, Natalie G. Trueman, Mark E. Burkard

**Affiliations:** 1 University of Wisconsin Carbone Cancer Center, University of Wisconsin-Madison, Madison, Wisconsin, 53705, United States of America; 2 Department of Medicine, Hematology/Oncology Division, University of Wisconsin-Madison, Madison, Wisconsin, 53705, United States of America; National Cancer Institute, NIH, UNITED STATES

## Abstract

Mitosis is coordinated by carefully controlled phosphorylation and ubiquitin-mediated proteolysis. Polo-like kinase 1 (Plk1) plays a central role in regulating mitosis and cytokinesis by phosphorylating target proteins. Yet, Plk1 is itself a target for posttranslational modification by phosphorylation and ubiquitination. We developed a chemical-genetic complementation assay to evaluate the functional significance of 34 posttranslational modifications (PTMs) on human Plk1. To do this, we used human cells that solely express a modified analog-sensitive Plk1 (Plk1^AS^) and complemented with wildtype Plk1. The wildtype Plk1 provides cells with a functional Plk1 allele in the presence of 3-MB-PP1, a bulky ATP-analog inhibitor that specifically inhibits Plk1^AS^. Using this approach, we evaluated the ability of 34 singly non-modifiable Plk1 mutants to complement Plk1^AS^ in the presence of 3-MB-PP1. Mutation of the T-loop activating residue T210 and adjacent T214 are lethal, but surprisingly individual mutation of the remaining 32 posttranslational modification sites did not disrupt the essential functions of Plk1. To evaluate redundancy, we simultaneously mutated all phosphorylation sites in the kinase domain except for T210 and T214 or all sites in the C-terminal polo-box domain (PBD). We discovered that redundant phosphorylation events within the kinase domain are required for accurate chromosome segregation in anaphase but those in the PBD are dispensable. We conclude that PTMs within the T-loop of Plk1 are essential and nonredundant, additional modifications in the kinase domain provide redundant control of Plk1 function, and those in the PBD are dispensable for essential mitotic functions of Plk1. This comprehensive evaluation of Plk1 modifications demonstrates that although phosphorylation and ubiquitination are important for mitotic progression, many individual PTMs detected in human tissue may have redundant, subtle, or dispensable roles in gene function.

## Introduction

In mitosis, posttranslational modifications (PTMs) are crucial for regulating protein function and degradation [1–5]. Mass spectrometry has identified a large set of mitotic posttranslational modifications [6–9], but functional annotation is sparse. Therefore, it is critical to develop efficient techniques to accurately interrogate PTM function. Towards this goal, we have thoroughly evaluated PTMs on polo-like kinase 1 (Plk1), a core regulator of mitosis using chemical genetic complementation.

Plk1 is an ideal target for analysis because it is essential and plays multiple roles in mitotic progression. Knockout of *PLK1* in mice results in embryonic lethality and, in human cells, failure of mitotic progression and proliferation [10,11]. Complete loss of Plk1 function arrests cells in prometaphase, yet it also plays roles in other mitotic stages. Specifically, Plk1 is involved in mitotic entry after DNA damage [12–14], centrosome separation [15–17], stabilizing kinetochore-microtubule attachments [15,16,18], removal of cohesin from sister chromatids [16,19], and in triggering cytokinesis [3,11,20,21]. Thus it is possible that distinct Plk1functions depend on specific PTMs.

**Fig 1:**
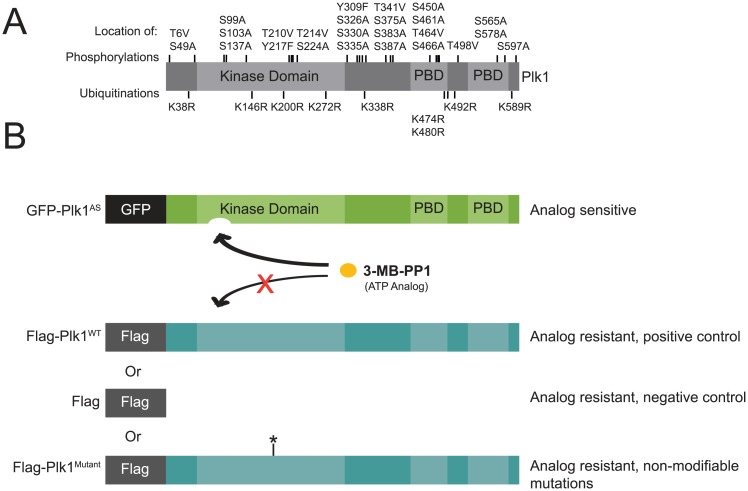
Using chemical genetics to assess the functional significance of Plk1 posttranslational modification sites. (A) Distribution of identified phosphorylation and ubiquitination sites on Plk1 by domain [8,22,23]. (B) Analog sensitive Plk1 (Plk1^AS^) is complemented with flag-tagged (analog resistant) rescue constructs. Flag-Plk1 is mutated (Plk1^Mutant^) to render it non-modifiable.

Here, we present a comprehensive strategy to evaluate the functional significance of PTMs on Plk1. We first evaluated databases of human Plk1 to identify 34 phosphorylation and ubiquitination modifications (Fig 1A) [8,22,23]. One crucial site is the activation loop phosphorylation on threonine 210. This site is phosphorylated by Aurora kinases A and/or B and the inability of cells to phosphorylate this residue leads to the Plk1-null phenotype [15,19,24–27]. Modifications at S137 and S326 have also been implicated in regulation of Plk1 functions. Phosphorylation at S137 increases the activity of Plk1 and is reduced in response to DNA damage [28,29]. Phosphorylation of Plk1 S326 promotes progression through mitosis [30]. Additionally, ubiquitination of K492 may be important for removal of Plk1 at the metaphase-anaphase transition [1]. However, the function of most posttranslational sites remains obscure.

To evaluate the function of the identified PTMs, We used non-modifiable mutant Plk1 to complement in a chemical genetic system. We employed previously established Plk1^AS^ cells as a chemical genetic tool to probe functions of Plk1 [11]. The analog-sensitive system is a versatile technique for studying kinases that provides a method for potent and reversible chemical inhibition with explicit controls for off-target effects [31]. In this system, GFP-tagged recombinant Plk1 (C67V/L130G) analog-sensitive (AS) mutant (GFP-Plk1^AS^; Fig 1B) was introduced into human hTERT-immortalized retinal pigment epithelial cells (RPE1) in which both endogenous alleles had been deleted. Plk1^AS^ is fully inactivated by 3-methylbenzyl pyrazolopyrimidine (3-MB-PP1) to reveal the Plk1 inhibition phenotypes including mitotic arrest and immature spindle poles.

Using this complementation assay, Plk1^AS^ cells were stably transduced with a second construct to express Flag-tagged Plk1 that harbors a wildtype kinase domain (Plk1^WT^) and is thus resistant to 3-MB-PP1, allowing for chemical genetic complementation [32] (Fig 1B). When challenged with 3-MB-PP1, the complementing wildtype Plk1 restores activity, allowing cells to complete normal mitosis [32]. We then introduced mutations into the Plk1 rescue construct to determine whether non-modifiable mutants can execute specific functions within mitosis. This system, chemical genetic complementation, allows for rapid temporal inhibition and we demonstrate that it can robustly identify functional PTMs on Plk1.

As we report here, most PTMs on Plk1 have redundant or subtle functions when evaluated singly. Only single PTMs in the kinase activating loop are essential for mitotic progression and long-term proliferation. Additional kinase domain sites operate redundantly. In contrast phosphorylation sites in the PBD are dispensable for essential Plk1 functions as a 16-site mutant restores viability. These data reveal the complexity of PTM-regulation of an essential mitotic protein wherein some PTMs are strictly required, some are redundant, and many are dispensable.

## Results

### Functional survey of PTMs on Plk1

**Fig 2:**
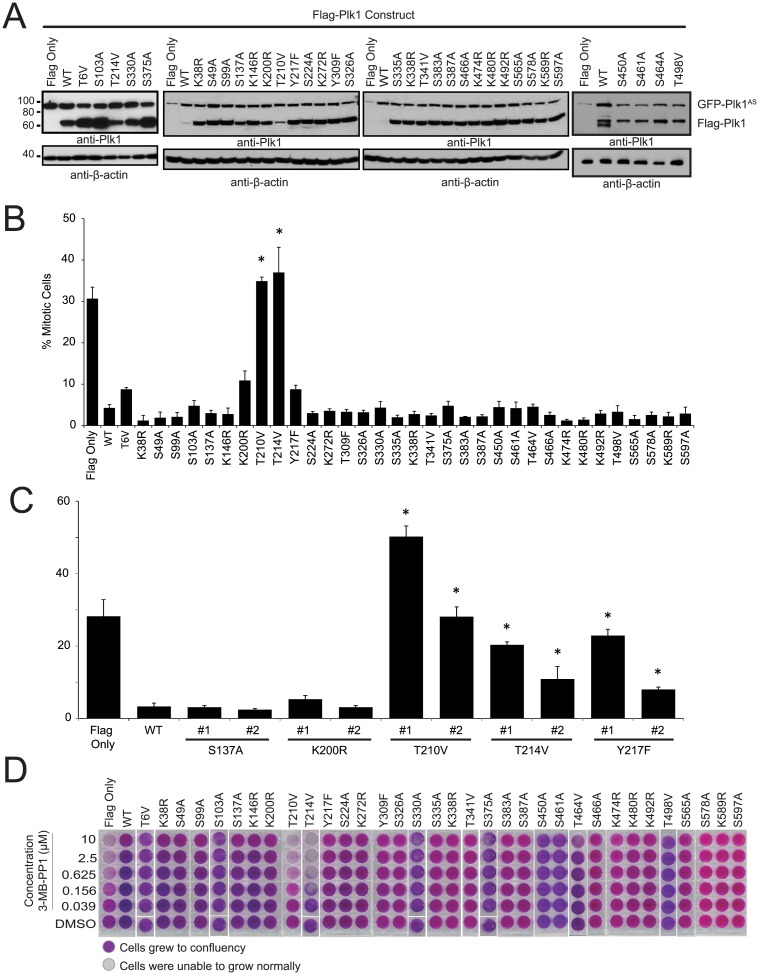
Plk1 T214V disrupts progression of mitosis and cell proliferation. (A) Western blots showing the expression of analog sensitive Plk1 (GFP-Plk1^AS^) and Flag-tagged Plk1 (Flag-Plk1) constructs with and without different S/T mutations in RPE cell lines. Cells were synchronized in mitosis with nocodazole prior to collection. Blot probed with anti-Plk1 (upper) and anti-β-actin (lower) antibodies. (B) Mitotic index of AS cell lines in (A) treated with 10μM 3-MB-PP1 for 16 hours. 900 cells were scored as mitotic or non-mitotic through Hoechst 33258 staining. n≥3, *p<0.05 compared to WT (GFP-Plk1^AS^/Flag-Plk1^WT^). (C) Mitotic index of Flag only and monoclonal AS cell lines in (S1A Fig) treated with 10μM 3-MB-PP1 for 16 hours. 900 cells were scored as mitotic or non-mitotic through Hoechst 33258 staining. n≥3, *p<0.05 compared to WT Clone (GFP-Plk1^AS^/Flag-Plk1^WT^). (D) 6000 cells were plated in complete medium and treated with concentrations of 3-MB-PP1 as indicated, and allowed to grow until control (DMSO) wells were confluent (six days). Cell density is qualitatively detected by crystal violet staining

To evaluate the functional significance of previously identified PTMs, we surveyed 34 mutations which prevent phosphorylation or ubiquitination of Plk1, selected from modification sites identified in proteomic analyses [8,22,23]. To do this, we stably expressed constructs each harboring a single non-modifiable mutation. Constructs were generally expressed at levels close to or greater than GFP-Plk1^AS^ (Fig 2A). To survey these for function, we challenged each cell line with 10 μM 3-MB-PP1 and evaluated for mitotic arrest (Fig 2B and 2C) and proliferation (Fig 2D). These two assays were selected because the mitotic arrest phenotype evaluates the function of Plk1 in assembling a mitotic spindle, whereas long-term proliferation requires Plk1 to execute its non-spindle mitotic functions. Additionally, hits in the mitotic arrest phenotype using polyclonal cell lines (Fig 2B) were re-evaluated in two clonal cell lines (Fig 2C and S1A Fig) to rule out artificial elevation in mitotic index through a sub-population of low-expressing cells.

As expected, mitotic arrest and proliferation defects occur in control (Flag Only), but not in cells rescued by Flag-Plk1^WT^, which is unaffected by 3-MB-PP1(Fig 2B–2D, leftmost bars or wells) [32]. Strikingly, most singly non-modifiable mutants of Plk1 restored mitotic progression and cell viability. A key exception is that bona fide mitotic arrest occurs with Plk1^T210V^, as noted previously, validating our assay [15,19,24–26]. Similarly, cells with Plk1^T210V^ are unable to proliferate over the long term in the presence of 3-MB-PP1 (Fig 2D).

In surveys of other mutations, we identified and confirmed defects in Plk1^T214V^, and to a lesser degree, Plk1^Y217F^, although the latter mutation was not essential for long-term proliferation (Fig 2D). Surprisingly, clonal Plk1^S137A^ is sufficient to rescue mitotic progression and proliferation, despite the known role of phosphorylation of this site in DNA damage and mitotic entry [26,28,29,33]. We conclude that while most single Plk1 modifications are non-essential under ordinary conditions, mutations at T210, T214, and to a lesser degree Y217 preclude the ability of Plk1 to execute its essential function for mitotic progression.

It is possible that some residual catalytic activity from GFP-Plk1^AS^ contributed to the rescue phenotypes. To evaluate this possibility, we employed a conditional knockout system to evaluate mutant Plk1 function in cells that do not express any other Plk1 allele. To do this, constructs were stably introduced into a Cre-sensitive *PLK1*^flox/Δ^ RPE1 cell line [11] (S1B Fig). Next, mitotic index was measured after challenging each with Cre recombinase to delete exon 3 of the floxed *PLK1* locus. Plk1^T210V^ and Plk1^T214V^ failed to rescue mitotic progression (S1C Fig), in contrast to other constructs. Likewise, clonal cell lines showed similar results upon Cre treatment (S1D and 1E Fig). Additionally, we subcloned cell lines after introducing Cre to determine if viable clones could be obtained. We recovered monoclonal *PLK1* ^Δ /Δ^ cell lines for S137A, K200R, and Y217F (S1F Fig). We conclude that PTMs of single sites, excepting T210 and T214, are dispensable for viability and mitotic progression in human cells.

### Functional evaluation of Plk1^T214V^

**Fig 3:**
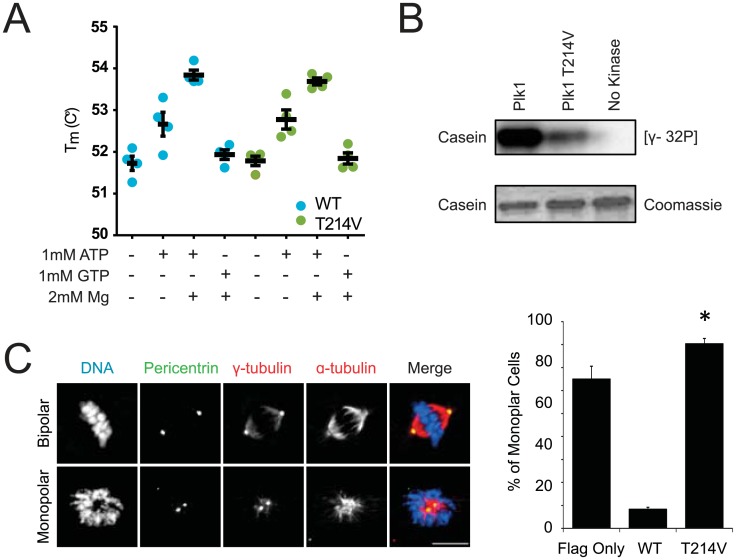
Plk1 T214V does not rescue Plk1 functions. (A) Plk1 T214V is not unfolded and can bind ATP. Recombinant His-tagged Plk1 kinase domains with and without T214V mutation were assessed for proper protein folding using Differential Scanning Fluorimetry. Equivalent conditions except where indicated. n = 3 (B) Recombinant His-tagged kinase domain of Plk1 with or without T214V mutation and α-casein were incubated with [γ-32P], resolved by SDS-PAGE, and visualized by autoradiography. (C) Cells shown were treated with 3-MB-PP1 for 6 hours, fixed, and stained with DAPI and for pericentrin, γ-tubulin and α-tubulin. n = 3, 300 or more cells were counted for each cell line. *p<0.05 compared to incidence of monopolar spindles in GFP-Plk1^AS^/Flag-Plk1^WT^ cells. Scale bar, 10μm.

Of the two functional Plk1 sites identified in our survey, T214 is novel. We considered whether function is impaired because of phosphorylation or if its hydroxyl group is important for these functions. To evaluate this, we first tested if T214 phosphorylation is detectable in mitotic cells. Multiple phosphoproteomic analyses have identified T214 phosphorylation [6,7,34]. Although we were unable to generate a phospho-specific antibody, mass spectrometry confirmed T214 phosphorylation on Plk1 immunoprecipitated from mitotic HeLa extracts (S2 Fig). To verify that mutation of T214 does not result in unfolding or prevent ATP binding, we employed differential scanning fluorimetry (DSF) [35]. Using recombinant Plk1 kinase domain, we examined melting temperatures in the presence and absence of ligand. Both Plk1^WT^ and Plk1^T214V^ had similar melting temperatures, indicating that the mutation does not disrupt Plk1 folding (Fig 3A). Moreover, both Plk1^WT^ and Plk1^T214V^ kinase domains are competent to bind ATP, as evidenced by temperature shifts upon ATP and Mg++ addition. These results are not an artifact of reaction conditions- no shift is detected with GTP and absorbance is absent without SYPRO or Plk1 protein (not shown). Further, Plk1^T214V^ retains some catalytic activity in an *in vitro* kinase assay with recombinant Plk1 (Fig 3B), indicating that T214 mutation does not completely abrogate kinase activity. Taken together, our findings confirm that T214 is phosphorylated and is required for Plk1 function, with no evidence that mutation merely interferes with kinase folding or ATP binding.

Because Plk1 has multiple roles in mitosis, we tested which particular function is impaired by failure to phosphorylate Plk1 at T214. Centrosome separation and bipolar spindle formation are early mitotic activities mediated by Plk1 [15,16]. As expected, Flag-Plk1^WT^ restored bipolar spindle formation whereas cells complemented with control (Flag Only) had a higher percentage of monopolar spindles (Fig 3C). Similar to Flag Only, Plk1^T214V^ failed to rescue bipolar spindle formation, demonstrating that the elevated mitotic index in these cells is mediated in part by impaired spindle assembly.

**Fig 4:**
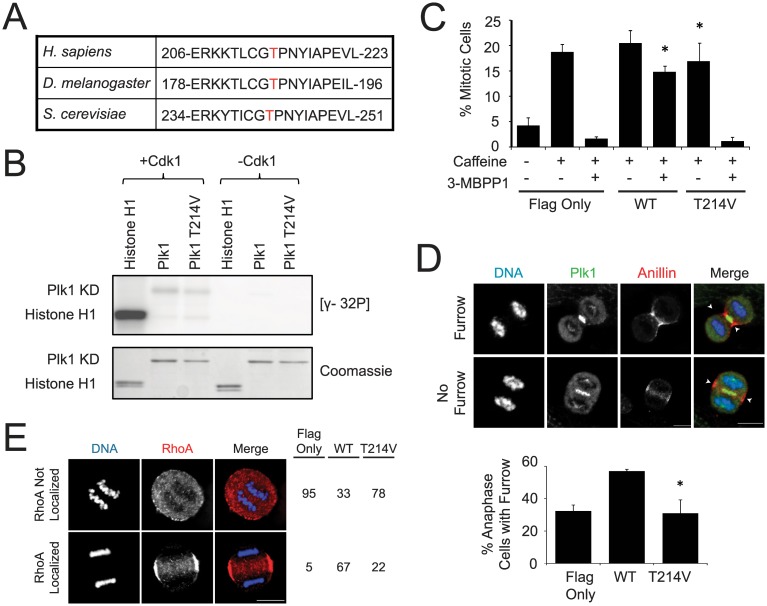
Plk1 T214 is not phosphorylated by Cdk1. (A) Sequence alignment of polo kinases with T214 or equivalent in red. (B) Recombinant Cdk1 and indicated substrates (His-tagged Plk1 kinase domains with or without T214V mutation or Histone H1) were incubated with [γ-32P], resolved by SDS-PAGE, and visualized by autoradiography. (C) Cell lines were synchronized with 5mM thymidine for 24 hours, released into complete media, treated with 0.2μg/ml doxorubicin for one hour, treated as indicated for 9 hours, stained with Hoechst, and scored as mitotic or non-mitotic. n = 3, 300 cells each, *p<0.05 compared to GFP-Plk1^AS^/Flag-Plk1^T214V^ cells treated with 5mM caffeine and 3-MB-PP1. (D) Cell lines were treated with 100μM monastrol for 8 hours, released for 40min, treated with 3-MB-PP1 for 20 min, fixed, and stained for Plk1, and Anillin and with DAPI. n = 3, >300 anaphase cells were quantified as having a furrow or not. *p<0.05 compared to incidence for furrow formation in GFP-Plk1^AS^/Flag-Plk1^WT^ cells. Scale bar, 10μm. Arrows indicate site of cleavage furrow. (E) Plk1 activity is required to localize RhoA to the equatorial cortex. Cells were synchronized with monastrol, released for 40 min, and then treated for 20 min with blebbistatin and 3-MB-PP1. Cells were scored for equatorial RhoA staining. n = 1, 100 cells per cell line were scored for RhoA localization.

We considered the upstream kinase mediating phosphorylation of Plk1 T214. Plk1 does not autophosphorylate at T214 (S3A Fig), so we examined the sequence flanking this phosphorylation to identify possible candidates. The +1 proline is conserved across species, and suggests phosphorylation by a proline-directed kinase, such as Cdk1 (Fig 4A). Furthermore, Cdk1 is a major mitotic regulator and its yeast homolog, cdc28, phosphorylates *S*. *cerevisiae* polo, Cdc5, at the homologous residue [36]. To test if Cdk1 activates human Plk1, we performed an *in vitro* kinase assay (Fig 4B). Cdk1 highly phosphorylated its substrate, Histone H1, but little phosphorylation was detected on purified Plk1 kinase domain, and this was not modulated by mutation at T214. We conclude that Cdk1 does not directly phosphorylate T214 of human Plk1.

The lack of Cdk1-dependent phosphorylation of T214 in our biochemical assay was surprising given the homology to *S*. *cerevisiae*; we therefore confirmed our findings with cell-based assays. Cdk1 activity markedly increases at mitotic onset and rapidly declines at anaphase [37–40]. If Cdk1 is required to activate Plk1, then Plk1 function will be impaired during DNA damage-recovery, before Cdk1 is activated [12,13] or during mitotic exit, when Cdk1 activity declines [3,21,41]. To test the former, we evaluated if Plk1^T214V^ promotes mitotic entry following DNA damage [12,13] and for the latter, we tested if Plk1^T214V^ can trigger cytokinesis furrow formation and RhoA localization to the equatorial cortex [3]. We found that Plk1^T214V^ fails to function on all accounts (Fig 4C–4E). Combined with our biochemical assay, we conclude that Cdk1 is not the upstream kinase of Plk1 T214. We considered additional proline-directed kinases, ERK1 and ERK2, but did not detect strong phosphorylation from either (S3B Fig), suggesting that neither are the upstream kinase for T214 phosphorylation. Although the upstream kinase of T214 phosphorylation remains elusive, we conclude that T214 is a crucial residue for mitotic functions of Plk1, although it remains possible that the hydroxyl is required for catalysis independent of its phosphorylation.

Because of T214’s proximity to the known Plk1 activation site, T210, we determined if T210 phosphorylation is contingent on intact T214. We found that T210 is phosphorylated in Plk1^T214V^ (S3C Fig). We conclude that T214 is a crucial residue for Plk1 function in mitosis, but is not upstream of T210 phosphorylation.

### Phosphorylations on Plk1 kinase domain have redundant functions

**Fig 5:**
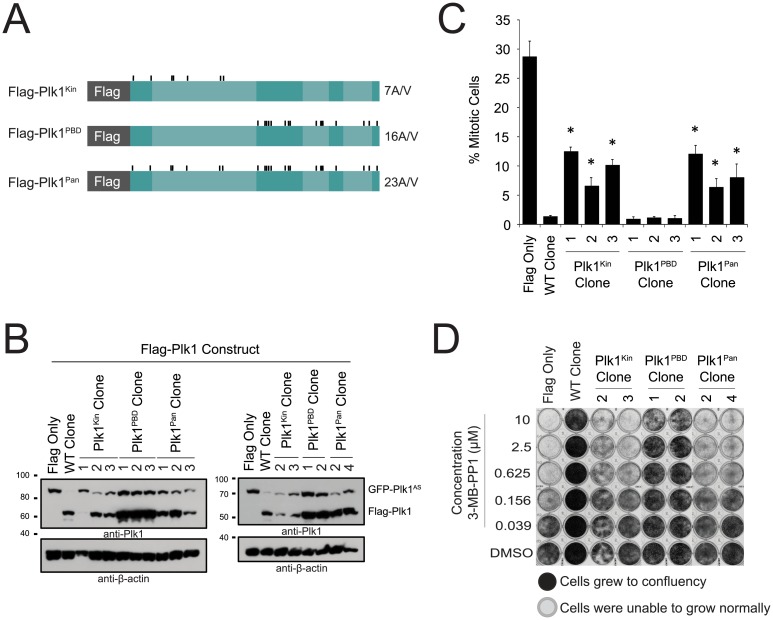
Plk1^PBD^ rescues mitotic progression and cell proliferation. (A) Plk1^AS^ is complemented with one of the indicated flag-tagged (analog resistant) rescue constructs. Flag-Plk1 is mutated at 23 phosphorylation sites (Plk1^Pan^, excluding T210 and T214) that were able to rescue in previous experiments. These 23 phosphorylation groups were then divided into two groups, based on domain. Plk1^Kin^ has 7 mutations in the kinase domain, while Plk1^PBD^ has 16 in the PBD region of the protein. (B) Western blot to show expression in indicated monoclonal cell lines probed with anti-Plk1 (upper) and anti-β-actin (lower) antibodies. (C) Mitotic index of AS cell lines from (B) treated with 3-MB-PP1 for 16 hours. 900 cells were scored as mitotic or non-mitotic through Hoechst staining. n≥3, *p<0.05 compared to WT Clone (GFP-Plk1^AS^/Flag-Plk1^WT^). (D) 6000 cells were plated in complete medium and treated with concentrations of 3-MB-PP1 as indicated, and allowed to grow until control wells were confluent (eight days). Cell density is qualitatively detected by crystal violet staining.

Most, individual PTMs surveyed in our complementation assays were not strictly required for viability of human cells. One possible explanation is redundancy of phosphorylation sites, in which phosphorylation at any one of several possible sites is sufficient to restore phosphorylation-dependent function. To evaluate this, we tested if simultaneous mutation of the 23 surveyed phosphorylation sites (Plk1^Pan^) abrogates Plk1 function (the 25 sites initially surveyed, without T210 or T214 mutation) or domain specific mutants (7 sites; Plk1^Kin^, 16 sites; Plk1^PBD^, Fig 5A and 5B). If the phosphorylation sites were redundant, simultaneous mutation of these sites would result in failure to restore essential functions of Plk1. Indeed, simultaneous mutation of all sites in Plk1^Pan^ resulted in elevated mitotic index and interfered with long-term proliferation of human cells as compared to Plk1^WT^ (Fig 5C and 5D), although these constructs localized properly in mitotic cells (S4A Fig). Surprisingly, Plk1^PBD^ restored both mitotic progression and cell proliferation, indicating that PBD phosphorylation sites are dispensable for the essential functions of Plk1. In contrast, Plk1^Kin^ is unable to restore mitotic progression or cell viability (Fig 5C and 5D), so redundant phosphorylation within the kinase domain is important for Plk1 function.

**Fig 6:**
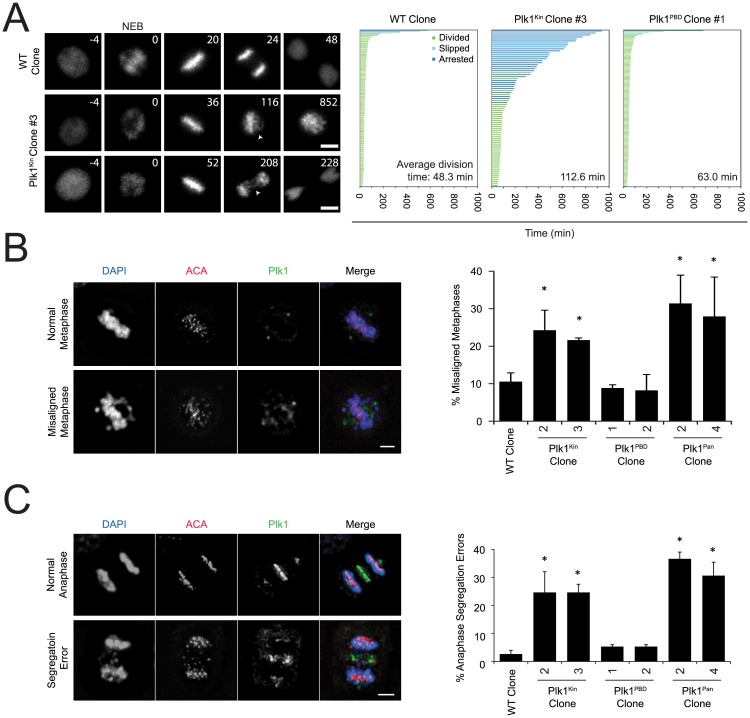
Plk1^Kin^ is unable to rescue chromosome alignment or chromosome segregation. (A) Cells were infected with H2B-FusionRed lentivirus for 48 hours, treated with 3-MB-PP1, and placed on microscope in chamber containing 5% CO_2_ at 37°C. Images were taken every 4min with a 20X objective for 16+ hours. Duration of mitosis was quantified from mitotic entry to when daughter cells sat down. Still frames from time lapse showing representative cell fates for WT and Plk1^Kin^ clones. Times indicate minutes from nuclear envelope breakdown (NEB). Arrows indicate examples of misaligned (middle row) and lagging chromosomes (bottom row). Scale bar, 5μm. Graph indicates individual cell fate and time in mitosis (≥ 79 cells in each condition). Average division time in lower right hand corner only includes cells that divided. Arrested (blue bars) indicates cells that did not divide during the movie. In this case, the data was censored and time in mitosis was quantified from mitotic entry to the end of the movie. Cells that began mitosis with <120min to the end of the movie and did not divide or left the screen were discarded. (B) Cells were treated with 3-MB-PP1 for 6 hours, fixed, and stained for Plk1 and ACA and with DAPI. n = 3, 63 or more metaphase cells were counted for each cell line for each replicate. *p<0.05 compared to incidence misaligned metaphases in WT Clone. Scale bar, 5μm. (C) Cells shown were treated with 3-MB-PP1 for 6 hours, fixed, and stained for DAPI, Antibody Against Centromere (ACA), and Plk1. n = 3, 60 or more anaphase cells were counted for each cell line for each replicate. *p<0.05 compared to incidence of anaphase segregation errors in WT Clone. Scale bar, 5μm.

To characterize the mitotic defect, cells were labeled with fluorescent H2B and imaged by timelapse videomicroscopy (Fig 6A). This revealed prolonged mitoses in the kinase domain mutant, characterized by congression defects and lagging chromosomes. In contrast, the Plk1^PBD^ 16-site mutant robustly restored mitosis with minimally changed mitotic duration. To characterize the mitotic defect further, we employed fixed cell analysis with indirect immunofluorescence. The Plk1^Kin^ mutant had increased frequency of prometaphase and metaphase cells, consistent with an early mitotic defect in spindle attachment (S4B Fig). However, these constructs were capable of separating centrosomes, an essential component of forming a bipolar mitotic spindle (S4C Fig).

We next characterized chromosome alignment. In the Plk1^Kin^ and Plk1^Pan^ lines, cells frequently had misaligned chromosomes on the metaphase plate (Fig 6B). Additionally, cells that proceeded into anaphase frequently had an increase in anaphase segregation errors (Fig 6C). These phenotypes were concordant between Plk1^Kin^ and Plk1^Pan^, but distinct from Plk1^PBD^, and consistently demonstrated the importance of redundant phosphorylation in the Plk1 kinase domain. Additionally, these phenotypes are consistent with chromosome-microtubule attachment errors, seen previously with defects in Plk1 kinase activity [42]. The Plk1^Kin^ has some reduced catalytic activity in an *in vitro* kinase assay relative to immunoprecipitated WT Plk1 (S4D Fig), suggesting that impaired catalytic activity may be, in part or wholly, responsible for this phenotype. We conclude that phosphorylation events within the kinase domain operate in a redundant manner, and are required for full Plk1 function.

## Discussion

Mass spectrometry has led to the discovery of a large number of PTMs on mitotic proteins [6–9]. However, functional assessment of PTMs can be challenging with commonly used techniques. In this study, we extend our chemical genetics system to evaluate the functional significance of 34 PTMs on a single mitotic protein, Plk1. Surprisingly, two sites are strictly and singly essential, there is redundancy among an additional 7 phosphorylation sites within the kinase domain, and 16 phosphorylation sites within the C-terminal polo-box domain are not required for essential functions of Plk1. This reveals that many observed mitotic PTMs are redundant, non-essential, or dispensable. If this is representative of PTMs on other mitotic proteins, then many observed PTMs will have subtle or dispensable functions.

We have identified T214 as a novel residue essential for Plk1mitotic functions. This is similar to that observed in yeast where the homologous residue is phosphorylated to promote mitotic functions of Cdc5 [36]. This site is required either because it is phosphorylated, or because the hydroxyl is required for catalysis exclusive of its phosphorylation state [43]. We and others have detected T214 phosphorylation in nocodazole treated cells, however it is unclear if it occurs during unperturbed mitosis. Supporting the former idea, phosphorylation on the equivalent residue has been observed in other mitotic kinases, including MPS1 and Aurora A [8,43]. However, we have been unable to identify the upstream kinase phosphorylating this site.

Surprisingly, most single phosphorylation sites on Plk1 are not required for its essential functions. However, the identification of redundant phosphorylation sites within the kinase domain has important implications. These redundant sites are essential for chromosome alignment in metaphase and maintenance of proper chromosome segregation during anaphase, which may be attributable to a moderate loss of catalytic activity [42]. This phenotype may also support a role of Plk1 in the spindle assembly checkpoint [44,45]. These mutations do not disrupt all Plk1 function, as rescue of centrosome separation was observed. Taken together, these data demonstrate that proper Plk1 function in mitosis requires specific phosphorylation of T-loop residues and one or more redundant phosphorylations in the kinase domain, but does not require PBD phosphorylation for its ordinary functions.

Among the mutations, functional rescue with Plk1^S137A^ was most surprising. Previous studies have demonstrated that S137 can be phosphorylated in mitosis [26,29], although levels of phosphorylation are low in HeLa cells [28]. Phospho-mimetic S137D increases Plk1 kinase activity and alters mitosis [26,28]. In U2OS cells, knockdown of endogenous Plk1 and replacement with Plk1^S137A^ results in metaphase arrest, suggesting that phosphorylation of this residue is important for metaphase-anaphase transition [26]. These are difficult to reconcile wholly with our observations here. However, the phosphomimetic findings do not indicate that S137 phosphorylation is necessary and S137A mutation does not reduce Plk1 activity [28]. Moreover, we do not exclude the possibility that S137 phosphorylation plays a critical role in the context of DNA damage. Similarly, we did not confirm a crucial role for K492. It has been suggested that ubiquitination of this residue is important for removal of Plk1 from the kinetochores [1]. In HeLa cells, knockdown of endogenous Plk1 and replacement with Plk1^K492R^ results in mitotic delay and an increase in apoptosis. The low penetrance of this phenotype may explain why we did not observe proliferation defects with K492R mutation. Alternatively there could be differences due to alternative models and methods used previously to deplete/replace endogenous Plk1. Some differences could be attributed to cell lines, since p53 null cells like Hela are more susceptible to Plk1 inhibition [46]. Our data suggest that S137 phosphorylation or K492 ubiquitination of Plk1 play important roles only for non-essential functions of Plk1.

There are some limitations of our observations. First, our assays are designed to observe Plk1 functions that are essential occur in differentiated human epithelial cells in mitosis. For example, our assays would not identify the effects of Plk1 phosphorylation of Orc2, which is not strictly required for S-phase [47]. Second, it is formally possible that the wildtype PBD of the Plk1^AS^ allele could complement in trans- with the second Plk1 allele. However, we find this unlikely as previous studies have shown the importance of a cis-acting PBD to localize Plk1 kinase activity [19,42,48], and we found concordant results using knockout for many mutants. Third, the polyclonal pools used for screening have varying expression levels. Although overexpression could cause mitotic defects, we derived subclones for mutants with strong mitotic defects making it unlikely that the defects were due to expression.

In conclusion, chemical genetics complementation can reveal the role of PTMs on Plk1. This system could be extended to other non-catalytic mitotic proteins using auxin-inducible degradation which is rapid and reversible [49]. Many other techniques, however, have limitations and the data can be difficult to interpret. RNA-interference based depletions may not sufficiently deplete endogenous protein and re-expressed non-modifiable mutants can be expressed heterogeneously by transient transfection. Residual levels of endogenous protein may impair phenotypic assessments—for example, 10% residual Plk1 is sufficient to complete mitosis, whereas knockout or >90% chemical inhibition reveals the null phenotype [11,42,50]. Genetic techniques can edit the genomic copy, but complex conditional systems are required for essential proteins. Moreover, these techniques lack the temporal resolution necessary to assess functions within the timescale of human mitosis of approximately one hour. Chemical genetic complementation, demonstrated here, provides allele specific, highly penetrant inhibition with the time advantage of a small molecule inhibitor to allow comprehensive analysis of PTM function. This can be applied to study function of other mitotic protein kinases.

## Materials and Methods

### Cell culture and synchronization

HeLa and hTERT-RPE1 (ATCC, Manassas, VA) cells were grown at 37°C in 5%CO_2_ in 1:1 mixture of DMEM and Ham’s F-12 medium supplemented with 2.5mM L-glutamine (hTERT-RPE1) or DMEM supplemented with 4 mM L-glutamine, 4500 mg/liter glucose (HeLa). Both were further supplemented with 10% fetal bovine serum and 100 units/ml penicillin-streptomycin.

Stable cell lines were created by retroviral infection. Recombinant viral particles were generated by cotransfecting Flag-tagged constructs in pQCXIN plasmid (Clontech) with vesicular stomatitis virus glycoprotein envelope plasmids into gag-pol expressing 293T Phoenix packaging cells using Fugene HD (Promega E2311). Target cells were treated with viral-containing media in the presence of polybrene. Cells expressing rescue construct were selected for using 0.4μg/ml G418. Cell lines for chemical genetic complementation were created from the EGFP-Plk1^as^ cell line used by Burkard et al. [11]. For experiments, cell lines selected by this method were used except when subcloned as indicated. For Cre-dependent knockout, constructs were created from flox/Δ cell line [11]. Clones were obtained by limiting dilution and selected using 0.4μg/ml G418.

All transient transfections were performed with HeLa cells and FuGENE HD transfection reagent with mitotic synchronization as noted. For anaphase synchronizations, cells were treated with monastrol for 8 hours, released for 40 minutes, treated for 20 minutes with 3-MB-PP1 +/- blebbistatin, and then fixed with 4% paraformaldehyde or 10% trichloroacetic acid. For Cre-dependent knockout of *PLK1*^flox/Δ^, cells were treated with Ad-Cre (Baylor University Vector Development Laboratory) at a multiplicity of infection of 5x10^4^ plaque-forming units per cell.

### Chemicals

Chemicals used in this study include 10μM 3-MB-PP1 (Toronto Research Chemicals), 5mM caffeine, 100μM monastrol (Tocris), 0.2μg/ml nocodazole (EMD Biosciences), 5mM thymidine (EMD Biosciences), 0.2μg/ml doxorubicin (MP Biomedicals), 50μM blebbistatin.

### Antibodies and Cell Stains

Antibodies used in this study include anti-phospho-210 PLK1 (BD 558400), anti-γ-tubulin (Thermo MA1-20248, clone GTU-88), anti-α-tubulin (MAB1864 Millipore), anti-anillin (polyclonal rabbit, Kim and Burkard, unpublished), anti-Plk1 (F-8, Santa Cruz Biotechnology sc-17783), anti-β-actin (AC-15, ab6276), anti-flag (M2) HRP (Sigma A8592), anti-RhoA (SC418 Santa Cruz), anti-pericentrin (ab44448 Abcam), anti-ACA (HCT0100 Immunovision), and anti-mouse HRP (Jackson Immunoresearch Laboratories Inc. #115-035-003). Immunoprecipitation of flag constructs was performed using anti-flag M2 affinity gel (Sigma A2220). For immunofluorescence, Alexa-flour antibodies were used (Invitrogen). Mitotic index was determined through Hoechst 33258 staining and microscopy. Crystal violet stain is composed of crystal violet (Sigma C-0775) with buffered formalin (Sigma HT-50-1-128)

### Recombinant Proteins

His-tagged constructs were cloned into pET-28a vector and kinase dead version has K82R mutation. Proteins were purified using Rosetta DE3 cells and extracted with Ni-NTA His-Bind Resin (Novagen, 70666). GST-tagged construct were cloned into pGEX-6P-1 vector. Proteins were purified using BL21 DE3 cells and extracted with Glutathione Sepharose 4B (GE Healthcare, 17-0756-01). Truncated protein included amino acids 1–352. Active Cdk1-Cyclin B (Invitrogen, PV3292), ERK1 (Promega, V1951), and ERK2 (Promega, V1961) were purchased as well as substrates Histone H1 (ab89813) and α-casein (Sigma C8032)

### Immunofluorescence, Microscopy, and Immunoblotting

For western blotting, cells were washed with PBS, incubated for 20 minutes on ice in lysis buffer (50mM HEPES, pH 7.5, 100 mM NaCl, 0.5% Nonidet P-40, 10% glycerol, 10mM sodium pyrophosphate, 5mM β-glycerol phosphate, 50mM NaF, 0.3mM Na_3_VO_4_,1mM phenylmethylsulfonyl fluoride (PMSF), 1X protease inhibitor mixture (Thermo Scientific), and 1mM dithiothreitol (DTT)), and centrifuged at 4°C. Equal amounts of protein were separated on SDS-PAGE, immunoblotted, and detected by chemiluminescence on film (Denville Scientific). Antibody incubations were performed in TBST + 4% milk or TBST + 5% BSA for phosphospecific antibodies.

For immunofluorescence (IF) cells were plated on coverslips. Antibody incubations were performed in PBS + 0.1% Triton X-100 with 3% BSA. Centrosome separation was determined following fixation with 100% ice cold methanol. Furrow formation was determined following fixation with 4% paraformaldehyde. RhoA accumulation at the equatorial cortex was determined following fixation with 10% trichloroacetic acid.

Image acquisition and analysis was performed on a Nikon Eclipse Ti inverted microscope with a CoolSNAP HQ2 charge-coupled device camera (Photometrics). Nikon Elements was used to process images which were transferred to Adobe Photoshop and Illustrator for final figures.

### Kinase Assays

All kinase assay reactions were incubated at 30°C for 30 minutes and resolved by SDS-PAGE. γ-^32^P incorporation was visualized by Typhoon TRIO imager (GE Healthcare). For Cyclin-dependent kinase 1, 100ng Cdk1-Cyclin B was incubated in buffer (50mM Tris-HCl, pH 7.5, 10mM MgCl_2_, 0.1mM NaF, 10μM Na_3_VO_4_) with 1mM DTT 1μM cold ATP, 5μCi [γ-^32^P] ATP, and 2μg substrate. For Plk1 autophosphorylation kinase assays, 100ng GST-Plk1 AA1-352 was incubated with concentration-matched indicated His-Plk1 substrate (His-tagged kinase dead Plk1kinase domain with or without T214V mutation), buffer (20mM Tris, pH 7.4, 10mM MgCl_2_, 50mM KCl), 100mM DTT, 1μM cold ATP, and 5μCi [γ-^32^P] ATP. For recombinant Plk1 kinase assays, His-tagged kinase domain of Plk1 with or without T214V mutation and α-casein were incubated with buffer (20mM Tris, pH 7.4, 10mM MgCl_2_, 50mM KCl), 100mM DTT, 1μM cold ATP, and 5μCi [γ-^32^P] ATP. For ERK kinase assays, ERK1 and ERK2 (Signal Chem, M29-10Uand M28-10G) were incubated in 1X Signal Chem buffer (K01-09) with 1mM DTT, 1μM cold ATP, 5μCi [γ-^32^P] ATP, and concentration-matched substrates.

Kinase activity of flag-tagged Plk1 was determined from stable cell lines. Flag constructs were immunoprecipitated with M2-agarose slurry and incubated in buffer (20mM Tris, pH 7.4, 10mM MgCl_2_, 50mM KCl) with 1mM DTT 1μM cold ATP, 5μCi [γ-^32^P] ATP, and 5μg α-casein.

### Mass Spectrometry

HeLa cells were transiently transfected with Flag-Plk1 and treated with nocodazole for 17 hours. Flag-Plk1 was immunoprecipitated with M2-agarose slurry and resolved by SDS-PAGE. Coomassie R-250 stained gel pieces were de-stained, dried, and rehydrated with 20μl of trypsin solution with 0.01% ProteaseMAX surfactant [10ng/μl trypsin (Trypsin Gold from PROMEGA Corp.) in 25mM NH4HCO3/0.01% w/v of ProteaseMAX (Promega Corp.)] The digestion was conducted for 3hrs at 42°C, peptides generated from digestion were transferred to a new Protein LoBind tube (~50μl volume) and digestion was terminated by acidification with 2.5% TFA [Trifluoroacetic Acid] to 0.3% final (7μl added). Supernatant was collected for spectrometry.

Peptides were analyzed by nanoLC-MS/MS using the Agilent 1100 nanoflow system (Agilent, Palo Alto, CA) connected to a hybrid linear ion trap-orbitrap mass spectrometer (LTQ-Orbitrap, Thermo Fisher Scientific, Bremen, Germany) equipped with a nanoelectrospray ion source. Chromatography of peptides prior to mass spectral analysis was accomplished using C18 reverse phase HPLC trap column (Zorbax 300SB-C18, 5μM, 5x0.3mm, Agilent) and capillary emitter column (in-house packed with MAGIC C18, 3 μM, 150x0.075mm, Michrom Bioresources, Inc.) onto which 8μl of extracted peptides were loaded. As peptides eluted, MS scans were acquired in the Orbitrap with a resolution of 100,000 and up to 5 most intense peptides per scan were fragmented and detected in the ion trap over the 300 to 2000 m/z; redundancy was limited by dynamic exclusion. Raw MS/MS data were converted to mgf file format using Trans Proteomic Pipeline (Seattle Proteome Center, Seattle, WA). Resulting mgf files were used to search against human Plk1 sequence. All of the predicted phosphopeptides were manually investigated to confirm proper phosphoresidue assignment.

### Differential Scanning Fluorimetry (DSF)

Recombinant Plk1 protein (5μg) was mixed with DSF buffer (400mM Hepes, 600mM NaCl, pH 7.5), 15X SYPRO, DMSO, and 5mM DTT. As indicated ATP (MP Biomedicals), GTP (Amersham Pharmacia Biotech Inc), and/or MgCl_2_ are added to the above mix and are run in 96 well plate in a RT-qPCR machine (Roche LightCycler^®^ 480 Instrument II) with the following protocol: Step 1: Temperature is increased 2°C per second to 25°C. Hold at 25°C for six seconds. Step 2: Temperature is increased 0.11°C per second to 95°C, acquiring five fluoresce measurements per degree; hold 95°C for 30 seconds.

### Genomic DNA

Genomic DNA was purified using Wizard SV Genomic DNA Purification System (Promega, A2360). Touchdown PCR was used to visualize the floxed locus using primers: AGGAAAGCCCTGACTGAGCC and TGCTTTTTACACAACTTTTGGGTTAC. Products were run on agarose gel and detected by ethidium bromide staining.

## Supporting Information

S1 Fig Probing the importance of Plk1 PTMs. (A) Western blots showing the expression in monoclonal analog sensitive Plk1 (GFP-Plk1^AS^) and flag-tagged Plk1 (Flag-Plk1) constructs with and without different S/T mutations in RPE cells. Blot probed with anti-Plk1 (upper) and anti-β-actin (lower) antibodies. (B) Western blot showing expression levels in indicated Cre-sensitive cell lines. Blots probed with anti-flag HRP (upper) and anti-β-actin (lower) antibodies. (C) Mitotic index of cells treated with Ad-Cre for ~48 hours. 900 cells were scored as mitotic or non-mitotic through Hoechst staining. n≥3, *p<0.05 compared to Plk1^flox/WT^ cell line. (D) Western blot showing expression levels in indicated monoclonal Cre-sensitive cell lines. Blots probed with anti-flag HRP (upper) and anti-β-actin (lower) antibodies. (E) Mitotic index of cells treated with Ad-Cre for ~48 hours. 900 cells were scored as mitotic or non-mitotic through Hoechst staining. n≥3. (F) Genomic DNA was isolated from Cre-sensitive cell lines were treated with Ad-Cre for ~48 hours and then subcloned into 96-well plates. PCR was run using primers flanking either side of the floxed locus. Upper band indicates PCR fragment containing floxed-Plk1. Lower band indicates Plk1 locus excision.
(EPS)

S2 Fig Mass spectrometry confirms phosphorylation of Plk1 T214. Flag-Plk1 was transfected into HeLa cells, which were synchronized in mitosis for collection. After immunoprecipitation, samples were processed as indicated in methods. MS/MS spectra of peptides corresponding to the T214 tryptic fragment are shown with b-ions in red and y-ions in blue. The table shows the expected m/z for ions observed.
(EPS)

S3 Fig Plk1 is not autophosphorylated or phosphorylated by ERK1/2. (A) GST-Plk1 and kinase dead (KD) His-Plk1 kinase domains, with and without T214V mutation, were incubated with [γ-32P], resolved by SDS-PAGE, and visualized by autoradiography. (B) ERK1 or ERK2 and His-Plk1KD kinase domain, with and without T214V mutation, were incubated with [γ-32P], resolved by SDS-PAGE, and visualized by autoradiography. (C) HeLa cells were transiently transfected with flag-tagged constructs and arrested with nocodazole for 5.5 hours. Cell extracts were incubated with anti-flag agarose beads. Pulldown samples were resolved by SDS-PAGE and blotted with anti-phospho-T210 antibody. At right, western blot showing input and immunoprecipitation (IP) controls. Blots probed with anti-flag antibody.
(EPS)

S4 Fig Plk1^Kin^ rescues some Plk1 functions. (A) Plk1^Pan^ localizes properly in early and late mitosis. Cells were stained for total Plk1, Flag, and ACA and with DAPI. (B) Cells were treated with 3-MB-PP1 for 6 hours, fixed, and stained for DAPI, ACA, and Plk1. n = 3, 200 or more mitotic cells were counted for each cell line for each replicate. (C) Cells were treated with 3-MB-PP1 for 6 hours, fixed, and stained for DAPI and γ-tubulin. n = 3, 60 or more cells were counted for each cell line for each replicate. *p<0.05 compared to incidence of not separated centrosomes in WT Clone. (D) Stable cell lines with indicated flag-tagged constructs were arrested with 0.2μg/ml nocodazole. Cell extracts were incubated with anti-flag agarose beads. Pulldown samples were incubated with [γ-32P] and α-casein, resolved by SDS-PAGE, stained with coomassie, and visualized by autoradiography. Western blot showing immunoprecipitation control probed with anti-flag antibody.
(EPS)

S1 Movie Normal mitosis in WT cell. (AVI)

S2 Movie Arrested mitosis in Plk1^Kin^ cell. (AVI)
